# Primary multiple endocrine insufficiency during immune checkpoint inhibitor treatment: A case report

**DOI:** 10.1097/MD.0000000000036998

**Published:** 2024-01-19

**Authors:** Yaning Wang, Peng Zhao, Ziyun Zhao, Hai Yang, Fanghua Zhang

**Affiliations:** a School of Clinical Medicine, Weifang Medical University, Weifang, Shandong, China; b Department of Endocrinology, Qingdao Central Hospital, Qingdao, Shandong, China; c Department of Laboratory, Qingdao Central Hospital, Qingdao, Shandong, China; d Department of Pharmacy, Qingdao Central Hospital, Qingdao, Shandong, China.

**Keywords:** hypoadrenalism, hypogonadism, hypothyroidism, immune-related adverse event, programmed cell death 1 inhibitor

## Abstract

**Rationale:**

Immunotherapy with immune checkpoint inhibitors (ICI) has shown promising activity against many tumor types. However, they can also induce a wide array of immune-related adverse events, ranging from mild to fatal. Primary 3 endocrine gland insufficiency during treatment with ICI has rarely been reported.

**Patient concerns:**

We report the case of a 33-year-old man with Ewing sarcoma who was treated with toripalimab as a second-line treatment. Approximately 11 months after initiating treatment, the patient developed subclinical hypothyroidism, which was followed by adrenal insufficiency and hypogonadism 6 months later. Consequently, the decision was made to discontinue ICI therapy and initiate hormone replacement therapy to manage endocrine deficiencies.

**Diagnoses:**

Serum adrenocorticotropic hormone, thyroid stimulating hormone, and prolactin levels increased significantly, while cortisol, estradiol, and testosterone levels decreased (Table 1). The patient had negative findings on the pituitary MRI.

**Intervention:**

As part of the management strategy, ICI therapy was ceased and hormone replacement therapy was commenced to address endocrine deficiencies.

**Outcomes:**

After hormone replacement therapy, his symptoms improved and follow-up examinations showed normalization of hormone levels.

**Lessons:**

Clinicians should be aware of the potential of immune checkpoint inhibitor therapy to cause endocrine dysfunction. Prompt recognition and management of these adverse events are crucial for patient health and quality of life.

## 1. Introduction

Immune checkpoint inhibitors (ICI) have shown promising antitumor activities against many tumor types. ICI release the inhibitory brakes of T cells, resulting in T-cell activation and robust antitumor immune responses.^[1,2]^ However, they can also induce a wide array of immune-related adverse events (irAEs) ranging from mild to fatal. Endocrinopathies are one of the most common irAEs, affecting up to 40% of patients receiving ICI. IrAEs can manifest as endocrinopathies involving the thyroid (hypothyroidism or thyrotoxicosis), pituitary (hypophysitis), adrenal gland (primary adrenal insufficiency), and pancreatic islets β-cell insulin-deficient diabetes, similar to type 1 diabetes.^[3]^ ICI-induced endocrinopathies are rarely fatal. But timely diagnosis and early intervention can considerably improve patient’s prognosis.

In the study, we report a 33-year-old man with Ewing sarcoma, who had multiple endocrine dysfunctions with subclinical hypothyroidism, hypoadrenalism, and hypogonadism after the treatment with toripalimab.

## 2. Case report

**Figure 1.**
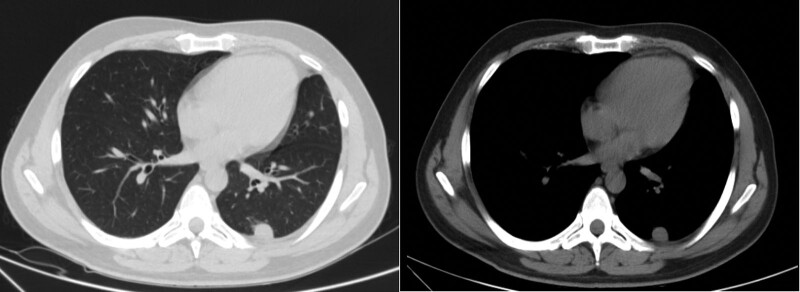
CT scans of patient: (A) and (B) pulmonary metastasis. CT = computed tomography.

**Figure 2.**
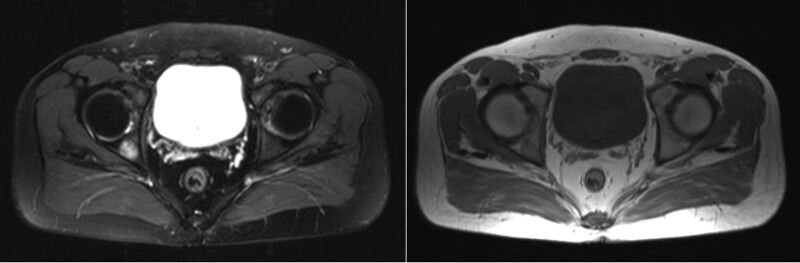
MRI scans of patient: (A) and (B) multiple bone and gluteal muscle metastasis. MRI = magnetic resonance imaging.

**Figure 3.**
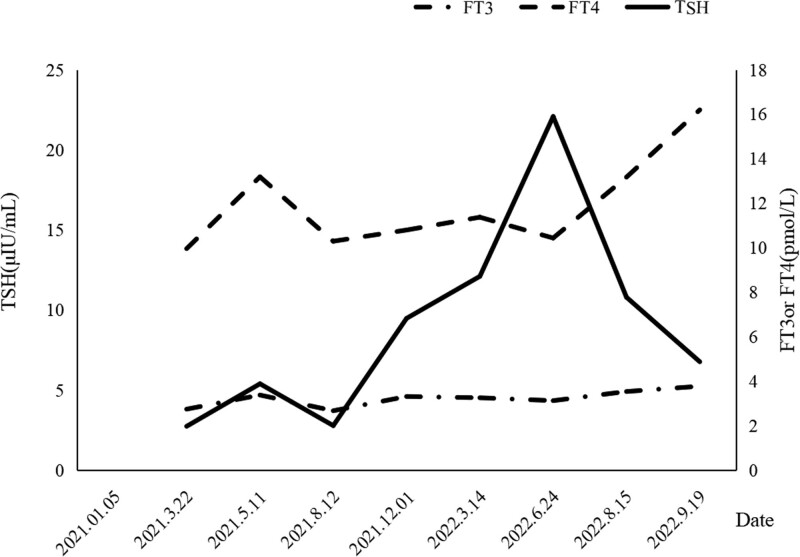
Longitudinal changes in TSH or FT3, FT4 over time and treatment for hypothyroidism. TSH = thyroid stimulating hormone.

A 33-year-old man was admitted to the hospital with chest pain for 4 months on December 23, 2016. Chest computed tomography (CT) revealed a chest wall mass and space-occupying lesion in the left upper lobe. Three days later, he underwent surgery, and histopathological analysis confirmed Ewing sarcoma of the chest wall without lymph node involvement (pathologic tumor node metastasis stage to T1N0Mx). He received 6 cycles of epirubicin plus cytoxan plus vindesine as adjuvant chemotherapy followed by radiation treatment for 27 times, and achieved complete response. In July 2019, a chest CT scan showed pulmonary metastasis from Ewing sarcoma of the chest wall (Fig. 1), suggesting recurrence. He received 6 cycles of gemcitabine plus docetaxel. In January 2021, he felt irritation in his hips, and MRI showed multiple bone and gluteal muscle metastasis (Fig. 2), suggesting disease progression. The patient received toripalimab 240 mg every 3 weeks. Levels of thyroid stimulating hormone (TSH), FT3, and FT4 were normal before August 12. In December 2021, he developed subclinical hypothyroidism (TSH 6.83 μIU/mL, FT3 4.61 pg/mL, FT4 15 ng/dL). After 3 months, hormonal follow-up examinations showed elevated TSH (8.72 μIU/mL) but normal FT3 and FT4 levels. Two months later, he was admitted with a sudden onset of fatigue, cold intolerance, appetite reduction, and decreased libido. On his physical examination, his body temperature was 36.3°C, blood pressure was 131/100 mm Hg, and heart rate was 100 bpm. Serum adrenocorticotropic hormone (ACTH), TSH, and prolactin levels increased significantly, while cortisol, estradiol, and testosterone levels decreased (Table 1). The patient had negative finding on the pituitary MRI. Laboratory workup and MRI confirmed adrenal insufficiency, subclinical hypothyroidism, and hypogonadism. The patient discontinued treatment with toripalimab as a result of grade 2 irAEs, and took prednisone (5 mg/d) and levothyroxine (25 µg/d) as a replacement. His fatigue resolved within a week. Two months later, levels of adrenocorticotropic hormone and TSH returned to 114.25 pg/mL and 7.78 μIU/mL, respectively. Longitudinal changes in TSH or FT3, FT4 over time and treatment for hypothyroidism in Figure 3. Until now, he was alive with stable disease after discontinuing toripalimab for a year, hormonal follow-up examinations showed that ACTH, prolactin, estradiol, testosterone, TSH, FT3, and FT4 level were normal.

**Table 1:** Biological investigation.

Endocrinology	
Value unit (reference value)	
Adrenocorticotropic hormone	155.69 pg/mL (7.0–65.0)
Cortisol	3.37 μg/dL (6.4–22.0)
Thyroid stimulating hormone	15.9 μIU/mL (0.27–4.20)
Free T3	4.35 pmol/L (3.1–6.8)
Free T4	14.50 pmol/L (12–22)
Antithyroglobulin antibody	<10.0 IU/mL (0–115.00)
Antithyroid peroxidase antibody	<9.00 IU/mL (0–34)
Thyrotropin receptor antibodies	<0.277 ng/mL (0–1.50)
Progesterone	0.12 µg/L (0–0.149)
Prolactin	631.7 μIU/mL (4.1–28.9)
Luteinizing hormone	4.72 mIU/mL (1.8–8.6)
Follicle stimulating hormone	6.93 mIU/mL (0–12.4)
Testosterone	0.68 ng/mL (2.80–8.00)
Estradiol	15.89 μg/day (27.1–52.2)

## 3. Discussion

Although programmed cell death 1 (PD-1) inhibitor therapy temporarily inhibits tumor growth, PD-1 binding with its ligands PD-L1 or PD-L2 suppresses T-cell activity by reducing fuel supply inhibition of the PI3K/Akt pathway to block glucose uptake^[4]^; unwanted consequences of their mechanisms of action lead to many adverse reactions. According to a recent study, the most frequent endocrine complication is thyroid dysfunction (30%), which appears to be more common with anti-PD-1 treatment and combination ipilimumab-nivolumab treatment than with anticytotoxic T-lymphocyte-associated protein 4 monotherapy or anti-PD-L1 monotherapy.^[5]^ Adrenal insufficiency (0.7–2.43%) is a rare but serious immune-related event that is commonly caused by PD-1 inhibitors.^[1,6]^ However, there have been no reports on the involvement of gonadal function.^[7]^ In the present study, the case developed multiple endocrine Insufficiency after ICI treatment. This is the first present case to report primary 3 endocrine gland insufficiency during immune checkpoint inhibitor treatment.

The time onset of irAEs is typically weeks to months following ICI initiation.^[8]^ Moreover, late-onset toxicity after ICI cessation is also possible.^[9]^ How to diagnose multiple endocrine insufficiency caused by PD-1 inhibitors is the focus. First, it is necessary to distinguish primary from secondary hormonal problems. Perhaps most importantly for preventing harm is recognizing that hypopituitarism often causes central hypothyroidism, secondary adrenal insufficiency, and secondary hypogonadism. The diagnosis of hypopituitarism: low ACTH with a low cortisol, low or normal TSH with a low FT4, hypernatremia and volume depletion with diabetes insipidus, and low testosterone or estradiol with low luteinizing hormone and follicle-stimulating hormone. What is more, consider MRI of the brain with or without contrast with pituitary/sellar cuts in patients with multiple endocrine abnormalities.

In the present case, irAEs occurred during treatment with toripalimab for 17 months. During PD-1 inhibitor treatment, the patient experienced severe fatigue, cold intolerance, appetite reduction, and decreased libido. Determining the origin of new symptoms in this patient is important: symptoms may result in cancer progression, side effects of treatments for secondary effects of PD-1 inhibitor therapy, or chemotherapeutics. Before initiating PD-1 inhibitor therapy, the patient received epirubicin, cytoxan, vindesine, gemcitabine, and docetaxel chemotherapy. There are no reports on the rise in multiple endocrine gland dysfunctions caused by these above drugs. The main presentations of the cancer progression were localized pain and no new symptoms. The most likely outcome is that the PD-1 inhibitor therapy caused, which depended on endocrinological inspection and imaging examination (CT or MRI).

The following analysis will clearly illustrate this point.^[10]^ In this case, a normal MRI of pituitary and the laboratory test results in Table 1 can exclude hypopituitarism leading to secondary endocrine dysfunction. Second, based on the patient’s clinical presentation and the TSH levels before and after PD-1 inhibitor treatment, it can diagnose hypothyroidism. Symptoms of adrenal insufficiency are nonspecific and include nausea, fatigue, anorexia, abdominal pain, and weight loss. Low early morning serum cortisol is abnormal and the concomitant presence of a high-serum ACTH is suggestive of primary adrenal insufficiency, while low-serum ACTH is suggestive of secondary adrenal insufficiency.^[11]^ The diagnosis of primary adrenal insufficiency can made when secondary adrenal insufficiency is excluded according to clinical features, biochemical evidence, and medical imaging of the case. Finally, the diagnostic criteria for gonadal dysfunction are similar to those for adrenal insufficiency mentioned above. The author inquired about the patient’s medical history, and the patient reported normal sexual function and reproductive organs before receiving PD-1 inhibitors but experienced a decrease in libido after using toripalimab. Normal follicle-stimulating hormone and luteinizing hormone levels and decreased testosterone levels findings collectively suggest gonadal dysfunction. Based on the aforementioned evidence, the patient was diagnosed with primary multiple endocrine insufficiency (including hypothyroidism, hypoadrenalism, and hypogonadism) caused by PD-1 inhibitors.

Treatment of irAEs depends on the organ system affected and the grade of toxicity according to the common terminology criteria for adverse events (CTCAE) classification. According to the CTCAE, the patient with grade 2 adverse effects should stop ICI and treat the effects until adverse effects abate.^[12]^ Hormone replacement therapy is the mainstay of treatment for endocrine irAEs. Prescribe thyroid hormone supplementation in symptomatic patients with any degree of TSH elevation or in asymptomatic patients with TSH levels that persist 10 mIU/L. Administration of levothyroxine started at 25 to 50 mg/day, with the dose adjusted according to serum TSH levels.^[13]^ Therapy for someone with suspected adrenal insufficiency is best to use prednisone (5–10 mg daily) or hydrocortisone (10–20 mg orally in the morning, 5–10 mg orally in early afternoon). Taper stress-dose corticosteroids down to maintenance doses over 5 to 10 days.^[13]^ In this case, the patient stops ICI therapy because of CTCAE grade 2 irAEs and treats with prednisone 5 mg/d and levothyroxine 25 µg/d. Since endocrine dysfunction in irAEs is irreversible in most cases, therapy must continue for each endocrine irAEs.^[14]^ And with gradual tapering and hormone replacement, immunotherapy can safely be resumed with close monitoring, except in rare cases of adrenal crisis or other life-threatening conditions. After the evaluation of the patient’s condition by experts in the oncology and endocrinology departments, as well as the patient’s frailty, he ultimately discontinues further therapy with immunotherapy.

## 4. Conclusion

ICI therapy is a powerful and promising new tool for cancer treatment. However, clinicians should be aware of the potential for immune checkpoint inhibitor therapy to cause various endocrine dysfunctions. Prompt recognition and management of these adverse events are crucial for patient health and quality of life. It is important to monitor patients for potential endocrine-related adverse events during immune checkpoint inhibitor therapy and provide appropriate treatment.

## Acknowledgments

The authors would like to thank the patient for participating in this study.

## Author contributions

**Conceptualization:** Yaning Wang, Fanghua Zhang.

**Data curation:** Yaning Wang, Ziyun Zhao.

**Investigation:** Fanghua Zhang.

**Methodology:** Peng Zhao.

**Resources:** Fanghua Zhang.

**Supervision:** Hai Yang, Fanghua Zhang.

**Validation:** Ziyun Zhao, Hai Yang.

**Writing – original draft:** Yaning Wang.

**Writing – review & editing:** Yaning Wang, Peng Zhao, Fanghua Zhang.
